# SARS-CoV-2 Surveillance in Indoor Air Using Electrochemical Sensor for Continuous Monitoring and Real-Time Alerts

**DOI:** 10.3390/bios12070523

**Published:** 2022-07-13

**Authors:** Fei Lu, Ozhan Gecgel, Ashwin Ramanujam, Gerardine G. Botte

**Affiliations:** Chemical and Electrochemical Technology and Innovation (CETI) Laboratory, Department of Chemical Engineering, Texas Tech University, Lubbock, TX 79409, USA; lufei_seu@hotmail.com (F.L.); ozhan.gecgel@ttu.edu (O.G.); aramanuj@ttu.edu (A.R.)

**Keywords:** rapid detection technique, SARS-CoV-2 surveillance, continuous air monitoring, SARS-CoV-2 air collection, electrochemical SARS-CoV-2 sensor, air sampling

## Abstract

The severe acute respiratory syndrome related coronavirus 2 (SARS-CoV-2) has spread globally and there is still a lack of rapid detection techniques for SARS-CoV-2 surveillance in indoor air. In this work, two test rigs were developed that enable continuous air monitoring for the detection of SARS-CoV-2 by sample collection and testing. The collected samples from simulated SARS-CoV-2 contaminated air were analyzed using an ultra-fast COVID-19 diagnostic sensor (UFC-19). The test rigs utilized two air sampling methods: cyclone-based collection and internal impaction. The former achieved a limit of detection (LoD) of 0.004 cp/L in the air (which translates to 0.5 cp/mL when tested in aqueous solution), lower than the latter with a limit of 0.029 cp/L in the air. The LoD of 0.5 cp/mL using the UFC-19 sensor in aqueous solution is significantly lower than the best-in-class assays (100 cp/mL) and FDA EUA RT-PCR test (6250 cp/mL). In addition, the developed test rig provides an ultra-fast method to detect airborne SARS-CoV-2. The required time to test 250 L air is less than 5 min. While most of the time is consumed by the air collection process, the sensing is completed in less than 2 s using the UFC-19 sensor. This method is much faster than both the rapid antigen (<20 min) and RT-PCR test (<90 min).

## 1. Introduction

Although it has been two years since the emergence of COVID-19, according to the Centers for Disease Control and Prevention, the seven-day moving average of new cases in the United States has still been around the 100,000-mark amidst a huge vaccination drive. This calls for a general surveillance of surroundings for the presence of SARS-CoV-2 virus. A pooled surveillance method is already being used for rapid screening of saliva samples from multiple people at the same time. The number of samples being pooled ranges anywhere from 5 to 64 samples [1,2,3] and the number of tests to be performed is reduced by up to 93% [4]. The sensitivity of these pooled samples is not compromised heavily and up to 96% sensitivity can still be achieved by pooled surveillance [3]. However, this pooled surveillance has only been implemented by far for saliva samples. This still means that currently there is no real-time guarantee if the air in a room is SARS-CoV-2 free.

The focus has predominantly been on the prevalence of aerosol transmission and related risks [5,6,7,8,9,10,11] when it actually has to be on the need for COVID-19 surveillance sensors to ensure a safe indoor environment. While pooled surveillance based on saliva sampling might help isolate the group at risk, it consumes time and aerosol generation is a potential consequence. Hence, a surveillance method specific for sampling aerosols is required for the indoor environment. Renninger et al. emphasize the need for surveillance testing, especially when affected individuals are asymptomatic, and successfully use dust as a tool for COVID-19 surveillance [12]. In another study, air in a hospital room was sampled at 200 L per minute using an AerosolSense air sampler to efficiently detect SARS-CoV-2 present in the air [13]. However, most of these surveillance techniques are based on reverse transcription polymerase chain reaction (RT-PCR), which, although effective, is time consuming, expensive, and not suitable for providing real-time alerts.

The risk that comes with the indoor airborne transmission is enormous and modeling studies are being performed to understand these risks and mitigate them to help curb the spread. A study analyzed the risk involved by having an infected person in an office of four people or a classroom of 25 students [14]. This study mentions that the risk in an office and classroom with passive ventilation is 47% and 92% respectively. The possibility of such high risk in an indoor environment with passive ventilation calls for a better understanding of airborne SARS-CoV-2 transmission. Even though the effects of aerosol transmission have been studied theoretically, it has been practically difficult to sample these aerosols continuously from the indoor environment and monitor for SARS-CoV-2 in real-time. Hence, the need for real-time sensors for judicious alerting of people indoors upon the influx of SARS-CoV-2 is the path forward for curbing the spread of SARS-CoV-2 at the earliest.

This paper presents a pragmatic and novel approach of real-time detection of SARS-CoV-2 in air samples using an Ultra-fast COVID-19 Diagnostic Sensor (UFC-19) [15,16]. In this method SARS-CoV-2 is collected from air and delivers SARS-CoV-2 virions in deionized water (DI) water. The water samples are analyzed for SARS-CoV-2 using the UFC-19, that enables detection of the virus within seconds [15,16]. Appropriate air sampling methods are crucial for effective air monitoring. All-glass impingers have already been investigated for sampling of viral aerosols, such as, influenza [17], and severe acute respiratory syndrome (SARS) [17]. However, liquid glass impingers are not efficient if virus particles are smaller than 1 μm such as the SARS and MERS coronaviruses, which are smaller than 200 nm in diameter [18,19]. Therefore, we explored two other air sampling methods, Liquid Spot Sampler (internal impaction) and Coriolis Sampler (cyclone based), in this investigation by detecting the SARS-CoV-2 virus in the collected samples using the UFC-19 sensor. Liquid Spot Sampler has already shown its applications in the collection of SARS-CoV-2 virus in a contaminated hospital room [20]. An environmental air sampler, Coriolis Sampler, provided by Bertin Corp can work at a high flow rate of up to 300 L/min, which is ideal for fast collection and detection of air contamination. The Coriolis micro sampler has already been utilized in evaluating SARS-CoV-2 contamination in a hospital during the COVID-19 pandemic in London [21]. The collection time and limit of detection (LoD) of these two test rigs are compared and discussed.

## 2. Materials and Methods

**Figure 1:**
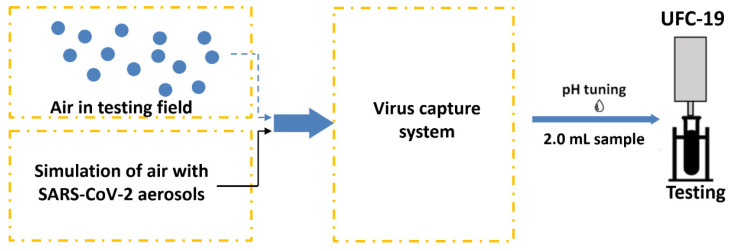
Test rig design capturing and testing SARS-CoV-2 in air. The system includes aerosolization of liquid samples into air to simulate the environment.

To simulate SARS-CoV-2 contamination and detection, test rigs were developed to generate SARS-CoV-2 aerosols, capture them, and detect the SARS-CoV-2 virus using the above-mentioned UFC-19 sensor. Different solutions with different SARS-CoV-2 concentrations were considered in the aerosolizers to determine the LoD. Figure 1 shows a schematic of the test rig where SARS-CoV-2 virions are aerosolized and captured in DI water. An aliquot of the SARS-CoV-2 containing water is used for testing in the UFC-19 after adjusting the pH.

### 2.1. Simulation of Air Samples Containing SARS-CoV-2 Virus

Heat inactivated SARS-CoV-2 virus (ATCC, VR-1986HK, Lot# 70042082, Manassas, VA, USA) was used as the target in this study. Heat inactivation was performed at 65 °C for 30 min followed by culturing the virus in Vero E6 cells for 14 days to ensure the prevention of virus replication. The inactivation and subsequent confirmation were both performed by ATCC before the product was received. To simulate the positive samples in air, heat inactivated SARS-CoV-2 virions were diluted to 32 cp/mL and further diluted to 10 cp/mL and 1 cp/mL for two different test rigs separately. These solutions were aerosolized and carried into the collector by the passthrough air. Two different aerosolizers were used: TSI Atomizer (TSI Incorporated, Shoreview, MN, USA) and PRONEB nebulizer (PARI Respiratory Equipment, Inc., Midlothian, VA, USA).

#### 2.1.1. TSI Atomizer

**Figure 2:**
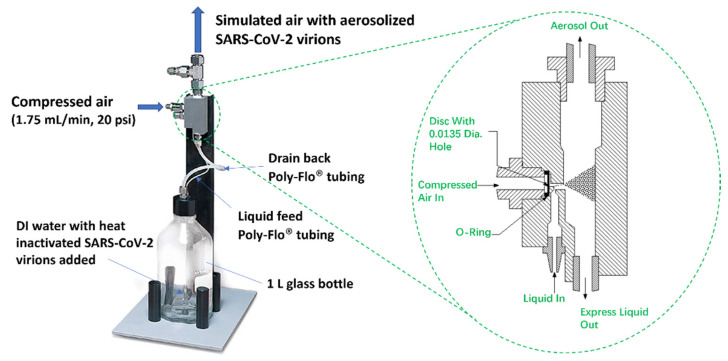
TSI MODEL 3076 Constant Output Atomizer used for aerosolizing heat inactivated SARS-CoV-2 virions. The SARS-CoV-2 containing solution was drawn into the atomizing section vertically through a Poly-Flo^®^ tubing. As shown in the zoomed-in figure of the atomizing section, the fine aerosolized spray left the atomizer through a silicone rubber tubing and flew into the air sampler while large droplets were removed by impaction on the wall opposite the jet and excess liquid was drained back into the glass bottle through another Poly-Flo^®^ tubing.

The heat-inactivated SARS-CoV-2 virions were aerosolized using TSI MODEL 3076 Atomizer as shown in Figure 2, which generated aerosols continuously for particles <1 µm [22]. A solution of 32 cp/mL heat-inactivated SARS-CoV-2 virions was prepared by diluting the stock in DI water. From this prepared solution, 250 mL was added in the 1L glass bottle and used as the source of the aerosolized virions. The solution was stirred continuously (Thermo Scientific Cimarec Stirring Hot Plate, 3 cm stirring bar, 180 rpm) to ensure uniform suspension of the virions during aerosolization. Compressed air (1.75 L/min, 20 psi pressure) flowed into the atomizer from an Ultra-Zero grade air gas cylinder (Airgas AI UZ300) through an orifice to form a high-velocity jet. The SARS-CoV-2 containing solution was drawn into the atomizing section vertically through a Poly-Flo^®^ tubing (provided by TSI Incorporated, Shoreview, MN, USA along with the TSI Atomizer Model 3076 Package) and then atomized by the jet. The fine aerosolized spray left the atomizer which was then guided with a Silicone Rubber tubing and flew into the air sampler for virion collection. In the meantime, large droplets were removed by impaction on the wall opposite the jet and excess liquid was drained back into the glass bottle through another Poly-Flo^®^ tubing.

To quantify the rate of aerosolization, at 1.75 L/min airflow and 20 psi pressure, the aerosolizer was operated for 13 h 30 min and the change in the solution volume in the aerosolizer bottle was monitored. In 4 repeated experiments, an average volume change of 45 mL of solution was observed, and an aerosolized flow rate of 55 μL/min output from the aerosolizer was obtained. The aerosolized heat-inactivated SARS-CoV-2 virions were captured using different methods explained in Section 2.2.

#### 2.1.2. PRONEB Nebulizer

**Figure 3:**
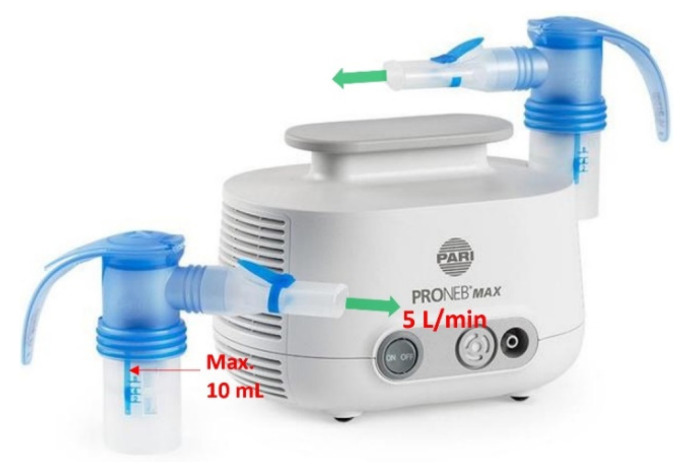
PRONEB nebulizer used to generate aerosols with heat inactivated SARS-CoV-2 virions in the test rig.

A PRONEB^®^ Max Nebulizer (PARI Respiratory Equipment, Inc., Model 130F35-LCS, Midlothian, VA, USA) shown in Figure 3 was implemented to simulate SARS-CoV-2 virions in air. This device requires a maximum of 10 mL solution to operate. The solution was aerosolized with a flow rate of 5.0 L/min (mixture of air and aerosol). In 50 min, the whole 10 mL of the solution was aerosolized, which corresponds to a flow rate of 0.2 mL/min output for the nebulizer, assuming a constant aerosolizing rate.

### 2.2. Air Sampling Methods to Capture SARS-CoV-2 Virions

Two air sampling methods were used to capture heat-inactivated SARS-CoV-2 virions: Liquid Spot Sampler™ (Aerosol Device Inc. Model LSS110A, Fort Collins, CO, USA) and Coriolis^®^ COMPACT sampler (Bertin Corp, Rockville, MD, USA).

#### 2.2.1. Liquid Spot Sampler

**Figure 4:**
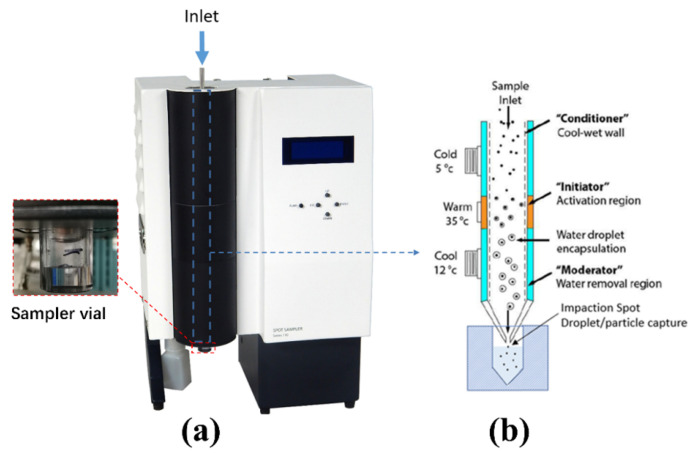
(**a**) Liquid Spot Sampler used for capturing SARS-CoV-2 virus, (**b**) capture mechanism of the Liquid Spot Sampler. The bio aerosol passed through the conditioner, initiator, and the moderator, grew to larger droplets, and was collected in the sampler vial through internal impaction.

The Liquid Spot Sampler™ shown in Figure 4a was used as one of the virus-capturing methods in this work. The Liquid Spot Sampler enlarges aerosol particles and then collects the particles into the liquid through internal impaction [23]. As shown in Figure 4b, the initial cold “conditioner” established a controlled vapor saturated aerosol stream largely independent of the incoming sample flow conditions. The warm walls of the “initiator” provided a region of high partial pressure of water vapor and generated supersaturation conditions. The final cool “moderator” region allowed continued droplet growth while reducing the flow temperature and water vapor content [23]. At a maximum flow rate of 1.75 L/min, the grown droplets were concentrated and collected in a 1 mL sampler vial at the bottom. The output of the aerosolizer was directly fed to the sampler from the inlet. The airflow was impacted upon the solution in the sampler vial to capture the virions. A 0.2 mL aliquot was taken from the vial and used for testing.

#### 2.2.2. Coriolis Sampler

**Figure 5:**
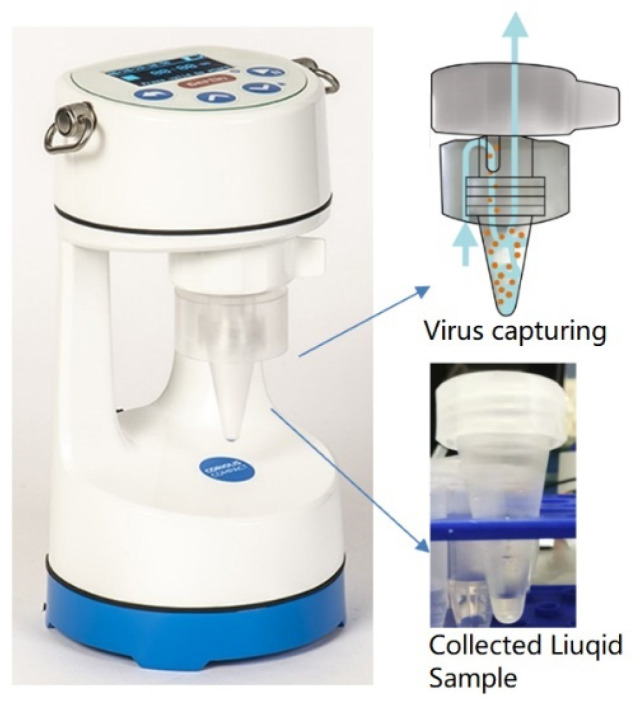
Coriolis Sampler used for capturing SARS-CoV-2 virus. Liquid samples containing SARS-CoV-2 virions were collected and centrifuged in a cone-shaped vial.

The Coriolis^®^ COMPACT sampler is a cyclone-based collector. It was used for collecting SARS-CoV-2 in air samples through a dry cyclonic technology with an airflow of 50 L/min [24]. Liquid samples were collected and centrifuged in a cone-shaped vial as shown in Figure 5. The Coriolis COMPACT is used for collecting the simulated air from the PRONEB Nebulizer due to its higher intake flow rate.

### 2.3. Ultra-Fast COVID-19 (UFC-19) Sensor

**Figure 6:**
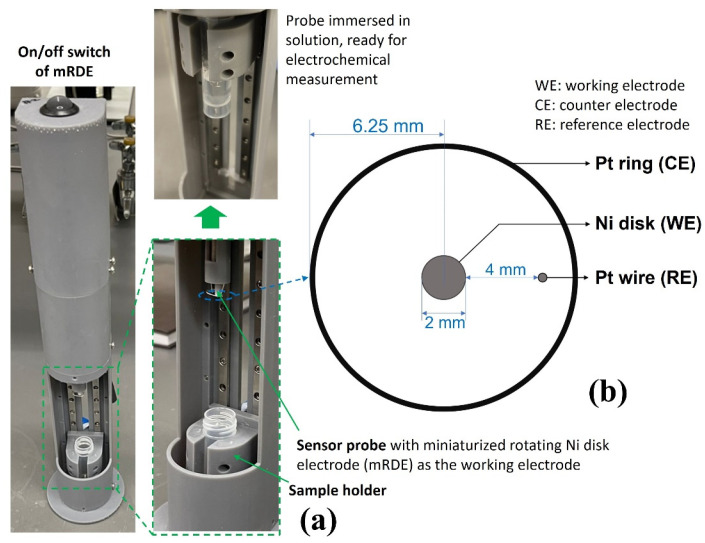
(**a**) UFC-19 Sensor used in experiments. The testing solution was put in the sample holder and slid up; the sensor probe was immersed in the testing solution for electrochemical sensing afterward. (**b**) Dimension of the electrodes of the UFC-19 sensor probe. The 3 electrodes were attached to Gamry Reference 600+ Potentiostat for electrochemical measurement.

The methodology of the UFC-19 sensor has been described in previous publications by Ramanujam and Botte [15,16]. In this work, a redesign of the three-electrode UFC-19 sensing device was implemented with a miniaturized rotating disk electrode setup (mRDE) as shown in Figure 6a. The body of the mRDE was printed with an SLA 3D printer (Form 3, FormLabs Inc., Somerville, MA, USA). The on/off switch at the top controls a DC motor of the mRDE that is attached to the Ni working electrode (McMaster-Carr Multipurpose 400 Nickel Rods ¼” diameter, one end machined to 2 mm diameter as the Ni working electrode). The sample holder, which holds the centrifuge tube, slides up and down to carry the sample solution to the sensing tip. The counter and pseudo reference electrodes were made of platinum wires 0.02” in diameter with a length of 2.75” and 1.25”, respectively (ESPI metals, Ashland, OR, USA; 3N5 purity), which can be seen in Figure 6b. The electrodes were attached to a Gamry Reference 600+ Potentiostat (Gamry Instruments, Warminster, PA, USA). Three 2.0-mL solutions were prepared in the centrifuge tube and used in the testing procedure, 1.0 M KOH solution as the “activation solution”, 0.01 M KOH solution as the “baseline solution”, and “testing solution” made by 0.2 mL collected liquid by the virus capture system and 1.8 mL 0.01 M KOH solution. The centrifuge tubes were placed in the sample holder (Figure 6a) and moved up to immerse working/counter/reference electrodes in the 2.0 mL solution for the measurement.

**Figure 7:**
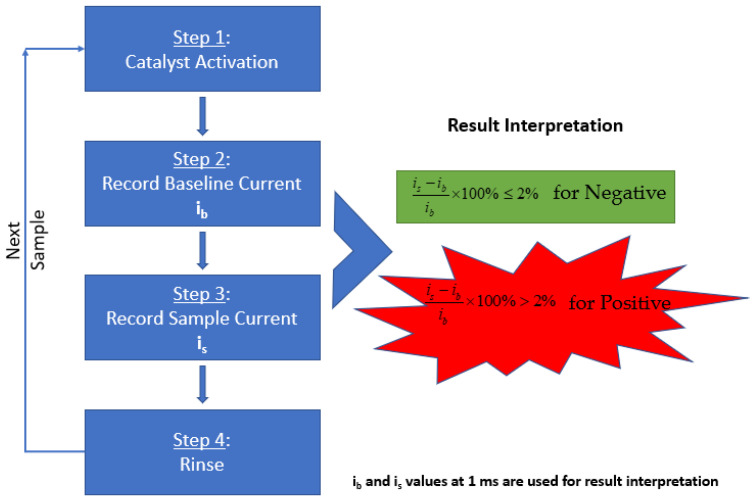
Schematic of the steps involved in diagnosing the aqueous sample solution using UFC-19. The baseline and sample currents recorded are processed through the mentioned equation to determine the result of the collected sample.

As mentioned in the previous publication by Ramanujam et al., the methodology includes a three-step process for SARS-CoV-2 detection performed with the Gamry Framework software [15]. In summary, as illustrated in Figure 7, the electrocatalyst was formed on the electrode surface by performing cyclic voltammetry in an activation solution. Following the formation of the catalyst, the baseline solution was tested by performing chronoamperometry to obtain the background signal. Next, the testing solution was tested under the same conditions to obtain the signal of the sample. The result was determined by comparing the baseline current and sample current.

To activate the catalyst, the sensor probe was preconditioned with 300 cycles of cyclic voltammetry (0.2 V to 0.6 V vs. Pt, 20 mV/s) using the “activation solution”. During the sensing process, three steps were operated. First, the “activation solution” was replaced by the “baseline solution” and the mRDE was rotated at 400 rpm, and one chronoamperometry experiment was performed by recording the oxidation current at a fixed oxidation potential of 0.58 V vs. Pt for 2 s. Second, the mRDE stopped rotating and the “baseline solution” was swapped by the “testing solution” in less than 20 s. Third, the mRDE rotation started again at 400 rpm, and the same chronoamperometry experiment was performed by recording the oxidation current at a fixed oxidation potential of 0.58 V vs. Pt for 2 s.

**Figure 8:**
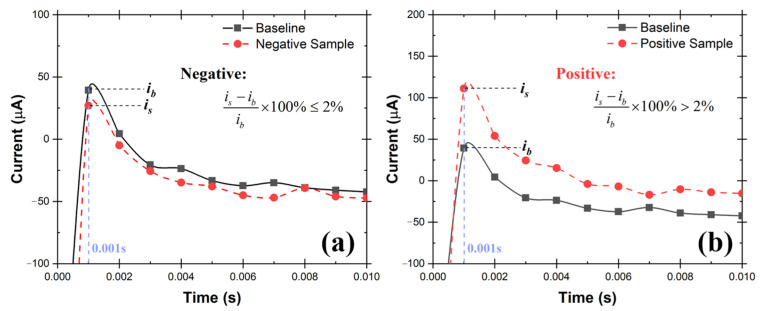
Example chronoamperometry graph of (**a**) Negative testing solution, (**b**) Positive testing solution. If the current at 0.001 s of testing solution is 2% higher than the baseline solution, the sample is Positive, otherwise, it is Negative.

The oxidation currents at 0.001 s of “baseline solution” (*i_b_*) was compared against the current of “testing solution” (*i_s_*) at the same time point. The criteria explained in Equations 1 and 2 were used to determine if the testing solution is Positive or Negative for SARS-CoV-2. The sample is determined to be Negative if the current response at 0.001 s point is equal or lower than 2% (Equation (1)). Otherwise, the sampled is determined to be Positive (Equation (2)). Examples of Positive and Negative testing solutions are shown in Figure 8a,b respectively. Before testing the collected samples, three known negative solutions (2.0 mL 0.01 M KOH in this case) were tested at the very beginning to make sure the catalyst activation was done properly to avoid false positive results.

(1) $$\mathrm{at}\;0.001\;s:\;\;\;\frac{i_{s}-i_{b}}{i_{b}}\times 100\%\leq 2\%,\;\mathrm{for}\;\mathrm{Negative}\;$$

(2) $$\frac{i_{s}-i_{b}}{i_{b}}\times 100\%>2\%,\;\mathrm{for}\;\mathrm{Positive}\;$$

UFC-19 focusses on detecting the spike protein S1 at a highly alkaline pH as mentioned in our previous work [15]. At such a high pH it becomes redundant if the virus is inactive or viable since extreme pH conditions are not suitable for virus viability. Therefore, it is expected that there should be minimal to no difference in detecting inactivated virus or a viable one by UFC-19.

## 3. Results and Discussion

### 3.1. Test Rig with TSI Atomizer and Liquid Spot Sampler

**Figure 9:**
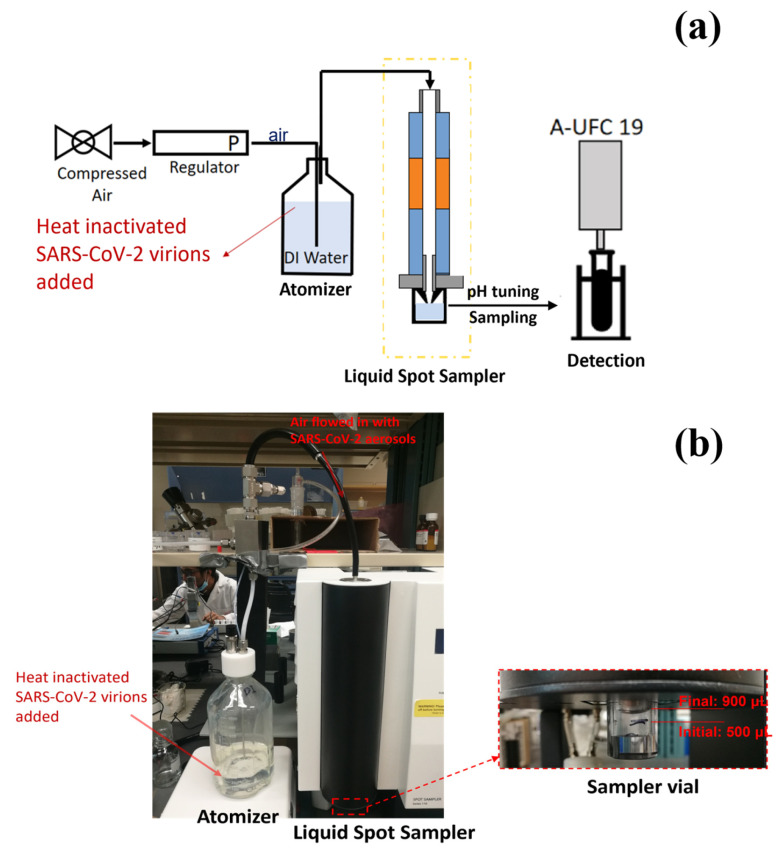
(**a**) Schematic of the test rig using TSI Atomizer and Liquid Spot Sampler. (**b**) Picture of the experimental setup used in the test rig.

**Figure 10:**
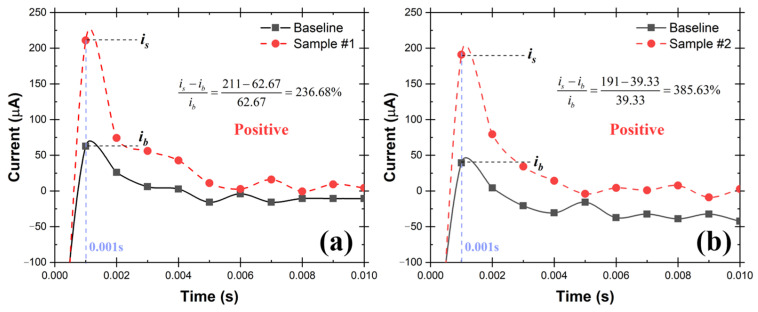
Chronoamperometry graph of the collected aqueous samples (3.67 cp/mL of SARS-CoV-2 virions) from two repeated air sampling experiments (**a**,**b**) using the test rig with TSI Atomizer and Liquid Spot Sampler. The current at 0.001 s of the collected samples is more than 2% higher than the baseline current indicating the samples are Positive.

A schematic of the testing system is shown in Figure 9, which consists of TSI atomizer, Liquid Spot Sampler, and the UFC-19 sensor. To simulate the SARS-CoV-2 in the air, the compressed air flowed into the TSI atomizer, containing 250 mL of 10 cp/mL SAR-CoV-2 solution in the bottle, and carried the SARS-CoV-2 aerosol out at a rate of 1.75 L/min, the maximum sampling flow rate of the Liquid Spot Sampler. Within 60 min, 0.9 mL SARS-CoV-2 containing solution was obtained, including the previously added 0.5 mL DI in the sampler vial. After that, 0.2 mL aliquot from the sampler vial was mixed with 1.8 mL commercial standardized pH 12 solution (LabChem, Potassium Hydroxide, 0.01 N, CAS # 1310-58-3) to make 2.0 mL of test solution. After performing the test, it can be seen from the chronoamperometry response graph in Figure 10 that the sample current response at 0.001 s is higher than the baseline by 236.68% and 385.63% respectively in the 2 demonstrated experiments. As mentioned before, this difference is greater than 2%, therefore both samples are determined to be Positive according to the criteria described in Section 2.3. The experimental conditions and testing results are shown in detail in Table 1. In 60 min, the sampler captured virus from 105 L of SARS-CoV-2 virus contaminated air. The limit of detection (LoD) in air for SARS-CoV-2 virions using this test rig is 0.029 cp/L. This was the lowest concentration that can be detected with 100% accuracy in 10 consecutive testing. In addition, the experiments provide evidence that the UFC-19 sensor can detect SARS-CoV-2 virus in a concentration of 3.67 cp/mL in an aqueous solution, which is significantly lower than the best-in-class assays (100 cp/mL) and FDA EUA RT-PCR test (6250 cp/mL) [25,26].

### 3.2. Test Rig with the Nebulizer and Coriolis Sampler

**Figure 11:**
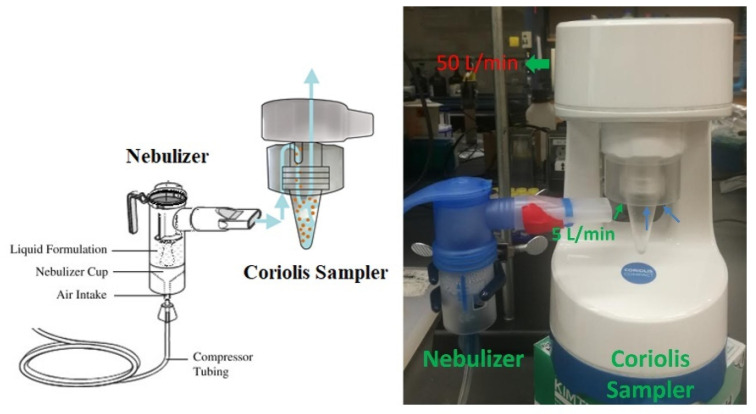
Simulation of SARS-CoV-2 in the air using PRONEB^®^ nebulizer and virus collection using the Coriolis COMPACT sampler. The cyclone-based sampler has a working flow rate of 50 L/min. During the experiment, 45.0 L/min of air was sucked with the 5.0 L/min of SARS-CoV-2 aerosols by the sampler at a flow rate of 50 L/min in total, leaving the liquid solution in the vial of the sampler.

**Figure 12:**
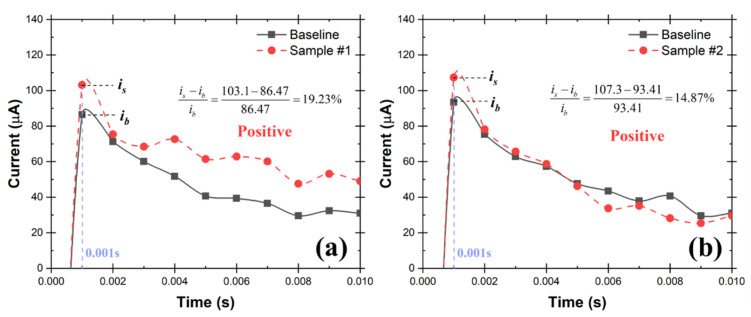
Chronoamperograms of UFC-19 sensor for collected samples (0.5 cp/mL SARS-CoV-2 virions) from two repeated air sampling experiments (**a**,**b**) using the test rig with Nebulizer and Coriolis Sampler. The current at 0.001 s of the collected samples is more than 2% higher than the baseline current indicating the samples are Positive.

In the test rig shown in Figure 11, a nebulizer was used to simulate SARS-CoV-2 in the air. The nebulizer has its own compressor which makes it convenient and inexpensive for aerosolization. An aqueous solution that contains 1.0 cp/mL SARS-CoV-2 virions was aerosolized at a rate of 0.2 mL/min by the nebulizer. The aerosols were carried out at a rate of 5.0 L/min and subsequently collected in the cone-shaped vial by the Coriolis COMPACT sampler. The testing solution was then made by diluting the 0.2 mL collected aqueous solution to 2.0 mL using commercial standardized pH 12 solution, which was then tested by the UFC-19 sensor. In two repeated experiments, the current increases at 0.001 s; the responsive chronoamperometry graph of the testing samples are 19.23% and 14.87% as shown in Figure 12, separately, which indicates that both samples are Positive according to the criteria. The experimental conditions and testing results are shown in detail in Table 2.

Fast air sampling is crucial for ultra-fast air monitoring. The experimental results in Table 2 show that the test rig using the Coriolis COMPACT Sampler is more sensitive when compared to the Liquid Spot Sampler. The LoD in the air is determined to be 0.004 cp/L in this test rig, which is 7.25 times lower than the one with Liquid Spot Sampler. This result was confirmed with 10 repeated experiments with the same results. In addition, the experimental results proved that the LoD of the UFC-19 sensor can be as low as 0.5 cp/mL, which is significantly better when compared to the best-in-class assays and RT-PCR test. Furthermore, the Coriolis COMPACT demonstrated a larger aspiration rate, it captured virus samples from 250 L of simulated air in 5 min. The sampled air could be enlarged to 1500 L in 5 min, using samplers with a larger aspiration rate such as the Coriolis Micro Sampler (300 L/min), making it more applicable for real-world applications. Previous research utilized the Coriolis Micro Sampler for 3 h using RT-PCR as the SARS-CoV-2 detection method. The LoD is 1.5 SARS-CoV-2 genome equivalents per 25 mL using real-time RT-PCR (translated to 60 cp/mL) [20]. In this investigation, with the utilization of the UFC-19 sensor, the LoD could be significantly reduced and therefore sampling time is reduced as well to achieve ultra-fast air monitoring.

It should be noted that the three steps of the sensing process took less than 24 s. Ideally, when everything is synchronized, the result can be given in 2 s after sample collection, which provides ultra-fast sensing faster than most of its counterparts such as the fast antigen (<20 min) [27] and RT-PCR (<90 min) test [28].

Furthermore, the current difference between the positive sample and the baseline (i.e., Figure 12) increases with higher expected SARS-CoV-2 concentration, similar to the sensor’s response to SARS-CoV-2 spike protein [15,26]. This allows for the possibility of developing a quantitative sensing method for SARS-CoV-2 virus particles using the UFC-19 sensor through linear regression.

## 4. Conclusions and Future Work

In this paper, two test rigs were developed to aerosolize, capture, and detect SARS-CoV-2 virions. The UFC-19 sensor was used as the SARS-CoV-2 detection method for airborne coronavirus sensing. The experiments demonstrated that the UFC-19 sensor can detect 0.5 cp/mL SARS-CoV-2 virions in aqueous solution. The detection limit is 200 times lower than the best-in-class assays, which provides flexibility in choosing air sampling methods. Both internal impaction and cyclone-based collection methods are feasible for airborne SARS-CoV-2 capturing, and the latter provides the lowest detection limit at 0.004 cp/L in air.

The test rig can collect testing samples from 250 L air in 5 min using the cyclone-based air capturing method. With the preconditioned UFC-19 sensor, the sensing time can be lower than 10 s, enabling ultra-fast SARS-CoV-2 sensing. The sensing time is lower than most of the sensing methods including the rapid antigen and RT-PCR test.

In the future, the test rig using the UFC-19 sensor will be calibrated and a quantitative method for detecting SARS-CoV-2 will be established. Investigating the specificity against common flu viruses such as H1N1, human CoV, etc., using the UFC-19 sensor is a priority. Machine learning will be integrated to develop a sensing algorithm that uses all data points in the chromatogram rather than currents only at 0.001 s to improve the specificity toward SARS-CoV-2. In addition, although in our testing for dust (Arizona test dust from Powder Technology Inc., Arden Hills, MN, USA.) contamination showed little to no effect in the SARS-CoV-2 detection, the tolerance of the sensor against environmental contaminations will also be investigated in more detail. Furthermore, our test rig will be validated by monitoring air in hospitals with SARS-CoV-2 infected patients to achieve our end goal of developing an ultra-fast and continuous air monitoring system for detection of SARS-CoV-2 and other airborne viruses. Finally, the placement of the sensors in the rooms will be optimized according to the location settings such as the size of the room, occupancy rate, and location of the doors and the vents.

## Figures and Tables

**Table 1:** Simulated air sensing with TSI atomizer and collection with the Liquid Spot Sampler.

Liquid Spot Sampler as the Virus Collector			Experiment #1	Experiment #2
**Air Flow**	Time	min	60	60
	Flow rate	L/min	1.75	1.75
	Air flowed	L	105	105
**Aerosol Generation (Input)**	Aerosolization rate	mL/min	0.0555	0.0555
	Aerosolized solution	mL	3.33	3.33
	SARS-CoV-2 virions concentration	cp/mL	10	10
	Virions left the atomizer	copies	33	33
**Simulated SARS-CoV-2 in Air**		**cp/L**	**0.31**	**0.31**
**Liquid Spot Sampler (Output)**	Vial solution	mL	0.9	0.9
	Expected concentration (Diluted 10 times in testing solution)	cp/mL	3.67	3.67
**Test Results**		**Positive or** **Negative?**	**True Positive**	**True Positive**

**Table 2:** Simulated air sensing with PRONEB nebulizer and collection with Coriolis Sampler.

Coriolis Sampler as the Virus Collector			Experiment #1	Experiment #2
**Air Flow**	Time	min	5	5
	Flow rate	L/min	50	50
	Air flowed	L	250	250
**Aerosol Generation (Input)**	Aerosolization rate	mL/min	0.2	0.2
	Aerosolized solution	mL	1.0	1.0
	SARS-CoV-2 virions concentration	cp/mL	1.0	1.0
	Virions left the nebulizer	copies	1	1
**Simulated SARS-CoV-2 in Air**		**cp/L**	**0.004**	**0.004**
**Coriolis Sampler (Output)**	Vial solution	mL	0.2	0.2
	Expected concentration (Diluted 10 times in testing solution)	cp/mL	0.5	0.5
**Test Results**		**Positive or** **Negative**	**True Positive**	**True Positive**

## Data Availability

Not applicable.
